# Challenges facing the United States of America in implementing universal coverage

**DOI:** 10.2471/BLT.14.141762

**Published:** 2014-09-23

**Authors:** Thomas Rice, Lynn Y Unruh, Pauline Rosenau, Andrew J Barnes, Richard B Saltman, Ewout van Ginneken

**Affiliations:** a Fielding School of Public Health, University of California-Los Angeles, 650 Charles Young Drive South, Los Angeles, CA 90095-1772, United States of America (USA).; b University of Central Florida, Orlando, USA.; c University of Texas-Houston, Houston, USA.; d Virginia Commonwealth University, Richmond, USA.; e Rollins School of Public Health, Emory University, Atlanta, USA.; f Berlin University of Technology, Berlin, Germany.

## Abstract

In 2010, immediately before the United States of America (USA) implemented key features of the Affordable Care Act (ACA), 18% of its residents younger than 65 years lacked health insurance. In the USA, gaps in health coverage and unhealthy lifestyles contribute to outcomes that often compare unfavourably with those observed in other high-income countries. By March 2014, the ACA had substantially changed health coverage in the USA but most of its main features – health insurance exchanges, Medicaid expansion, development of accountable care organizations and further oversight of insurance companies – remain works in progress. The ACA did not introduce the stringent spending controls found in many European health systems. It also explicitly prohibits the creation of institutes – for the assessment of the cost–effectiveness of pharmaceuticals, health services and technologies – comparable to the National Institute for Health and Care Excellence in the United Kingdom of Great Britain and Northern Ireland, the *Haute Autorité de Santé* in France or the Pharmaceutical Benefits Advisory Committee in Australia. The ACA was – and remains – weakened by a lack of cross-party political consensus. The ACA’s performance and its resulting acceptability to the general public will be critical to the Act’s future.

## Résumé

En 2010, juste avant que les États-Unis d'Amérique aient mis en œuvre les principales caractéristiques de la loi *Affordable Care Act* (ACA, loi sur les soins abordables), 18% des résidents des États-Unis d'Amérique âgés de moins de 65 ans de disposaient d'aucune assurance-maladie. Aux États-Unis d'Amérique, les insuffisances dans la couverture maladie et les modes de vie malsains contribuent aux résultats qui sont souvent comparés de manière défavorable avec les résultats observés dans les autres pays à revenu élevé. En mars 2014, l'ACA a considérablement modifié la couverture maladie aux États-Unis d'Amérique, mais il reste encore beaucoup à faire concernant la plupart de ses caractéristiques principales - échanges d'assurance-maladie, développement du *Medicaid*, création d'organisations de soins responsables et surveillance accrue des compagnies d'assurances. L'ACA n'a pas introduit les contrôles rigoureux des dépenses qui existent dans de nombreux systèmes de santé européens. Elle interdit également explicitement la création d'instituts – pour l'évaluation du rapport coût-efficacité des produits pharmaceutiques, des services et des technologies de santé – comparables au *National Institute for Health and Care Excellence* du Royaume-Uni de Grande-Bretagne et d'Irlande du Nord, à la Haute Autorité de Santé en France ou au *Pharmaceutical Benefits Advisory Committee* en Australie. L'ACA était – et reste – affaiblie par le manque de consensus entre les partis politiques. La performance de l'ACA et son acceptabilité par le grand public seront déterminantes pour l'avenir de la loi.

## Resumen

En 2010, inmediatamente antes de que los Estados Unidos aplicaran características clave de la Ley de Cuidado de la Salud Asequible (ACA, por sus siglas en inglés), el 18 % de los residentes de Estados Unidos menores de 65 años carecían de seguro de salud. En los E.E.U.U., las brechas en la cobertura de salud y los estilos de vida insanos contribuyen a unos resultados que a menudo son peores que los observados en otros países con ingresos altos. En marzo de 2014, la ACA modificó sustancialmente la cobertura de salud en los Estados Unidos, pero la mayoría de sus características principales, es decir, el intercambio de seguros médicos, la expansión de Medicaid, el desarrollo de organizaciones de atención médica responsable y la mayor supervisión de las compañías de seguros son aún tareas pendientes. La ACA no introdujo controles de gastos estrictos como los presentes en muchos sistemas de salud europeos. Además, prohíbe explícitamente la creación de institutos para la evaluación de la rentabilidad de productos farmacéuticos, servicios y tecnologías de la salud, similares al Instituto Nacional de Salud y Excelencia Clínica en el Reino Unido de Gran Bretaña e Irlanda del Norte, la *Haute Autorité de Santé* en Francia o el Comité Asesor de Beneficios Farmacéuticos en Australia. La aplicación de la ACA era (y sigue siendo) insuficiente por la falta de consenso político entre todos los partidos. El cumplimiento de la ACA y su aceptación consiguiente por la población general serán decisivos para el futuro de la ley.

## ملخص

في عام 2010، قبل تنفيذ الولايات المتحدة الأمريكية للملامح الرئيسية لقانون الرعاية ميسورة التكلفة مباشرة، كانت نسبة 18 **  **% من سكان الولايات المتحدة الذين تقل أعمارهم عن 65 سنة تفتقر إلى التأمين الصحي. وتسهم الثغرات في التغطية الصحية وأنماط الحياة غير الصحية، في الولايات المتحدة الأمريكية، في حصائل غالباً ما تتم مقارنتها على نحو غير مقبول بتلك التي يتم ملاحظتها في البلدان الأخرى مرتفعة الدخل. وبحلول آذار/ مارس 2014، غيّر قانون الرعاية ميسورة التكلفة بشكل كبير التغطية الصحية في الولايات المتحدة الأمريكية ولكن تظل معظم ملامحه الأساسية قيد التنفيذ- التبادل بين التأمين الصحي وتوسيع Medicaid وتطوير مؤسسات الرعاية القابلة للمساءلة وتعزيز الرقابة على شركات التأمين. ولم يقدم قانون الرعاية ميسورة التكلفة ضوابط الإنفاق الصارمة الموجودة في العديد من النظم الصحية الأوروبية. كما أنه يحظر صراحة إنشاء المؤسسات - بغرض تقييم مردودية المستحضرات الصيدلانية والخدمات الصحية والتقنيات - التي تضاهي المعهد الوطني للصحة وتميز الرعاية في المملكة المتحدة لبريطانيا العظمى وأيرلندا الشمالية، أو Haute Autorité de Santé (الهيئة العليا للصحة) في فرنسا أو اللجنة الاستشارية للفوائد الصيدلانية في أستراليا. وقد عاني قانون الرعاية ميسورة التكلفة- ولا يزال – من الضعف بسبب الافتقار إلى التوافق السياسي بين الأحزاب. وسوف يكون أداء قانون الرعاية ميسورة التكلفة وقبوله الناتج لدى عامة الشعب أهمية حاسمة بالنسبة لمستقبل القانون.

## 摘要

2010年，就在美国实施可负担医疗法案（ACA）的主要功能之前，在65岁以下的美国居民中，尚有18%居民缺乏健康保险。在美国，健康保险的缺口和不健康的生活方式产生的结果与其他高收入国家的情况相形见绌。到2014年3月，ACA已经在实质上改变了美国医疗保险状况，但其最主要的功能（医疗保险交易所、医疗补助计划扩张、发展负责的保健组织和进一步监督保险公司）尚未完全实现。ACA没有引入在许多欧洲卫生系统广泛采用的严格开支控制。它也明确禁止建立类似英国的国家卫生和护理卓越研究所、法国的健康管理局或澳大利亚的医药福利咨询委员会这样的机构来评估医药成本效益、卫生服务和技术。由于缺乏跨党派的政治共识，ACA（一直）受到削弱。对于法案的未来，ACA的绩效以及一般公众由此对其接受的程度至关重要。

## Резюме

В 2010 году, непосредственно после реализации ключевых положений Закона о доступном медицинском обслуживании (Affordable Care Act, ACA), медицинское страховое покрытие отсутствовало у 18% жителей США в возрасте до 65 лет. Отсутствие медицинского страхового покрытия вкупе с нездоровым образом жизни приводит к тому, что по ряду показателей США часто уступают другим странам с высоким уровнем доходов. К марту 2014 года закон ACA существенно изменил медицинское страховое покрытие в США, однако большая часть его основных положений — биржи медицинского страхования, расширение программы Medicaid, создание ответственных организаций по оказанию медицинской помощи и дополнительный надзор за страховыми компаниями — внедрены лишь частично. Закон ACA не предусматривает создание жестких механизмов контроля расходов, присущих многим европейским системам здравоохранения. Он также недвусмысленно запрещает создание институтов оценки экономической эффективности лекарственных препаратов, медицинского обслуживания и технологий подобных Национальному институту здоровья и клинического совершенствования в Соединенном Королевстве Великобритании и Северной Ирландии, *Haute Autorité de Santé* во Франции и Консультативному совету по лекарственному обеспечению в Австралии. По закону ACA отсутствовал – и до сих пор отсутствует – межпартийный политический консенсус. Эффективность и вытекающее из этого принятие общественностью закона ACA будут решающими для будущего закона ACA.

## Introduction

The Patient Protection and Affordable Care Act – commonly known as the Affordable Care Act (ACA) or Obamacare – was signed into law in 2010. The Act’s first open enrolment period – which began in October 2013 – was fraught with controversy because of severe problems with the web-based enrolment system and the popular realization that millions of people would not be allowed to renew their existing, nonconforming insurance policies. We deemed the end of the first open enrolment period for most United States’ residents – 31 March 2014 – to be an opportune time to assess the ACA’s impact and identify remaining challenges.

## Health system performance in the United States

In 2012, the United States of America (USA) spent more than 2.8 trillion United States dollars (US$) – i.e. more than 17% of its gross domestic product (GDP) and more than the entire GDP of the United Kingdom of Great Britain and Northern Ireland – on its health-care system.^1^^,^^2^ This spending meant that, in 2012, health-care expenditure per capita was substantially higher in the USA than in any other country. It was, for example, 50% higher than that in Norway – i.e. the Organisation for Economic Co-operation and Development (OECD) country with the next highest health-care expenditure per capita.^3^ Despite such spending on health care, many United States’ residents had no health insurance and several aggregate measures of health quality and outcomes recorded in the USA were poorer than the corresponding data from other high-income countries.^4^ Immediately before the implementation of the key elements of the ACA in 2014, 18% of residents younger than 65 years lacked any form of health insurance.^5^

As uninsured residents have relatively poor access to the offices of private physicians, they frequently seek care from so-called safety-net providers – such as community centres and the outpatient or emergency departments of hospitals. Compared with their counterparts in other high-income countries, patients in the USA are much more likely to forgo medications and to skip care – especially preventive care – because of costs.^6^

Gaps in health coverage, problems with access to health care and unhealthy lifestyles are thought to contribute to the many disappointingly poor health outcomes recorded in the USA. The USA performs better than most high-income countries in terms of breast and colorectal cancer survival and 30-day mortality rates for acute myocardial infarction and ischaemic stroke – probably because of high rates of screening for these conditions or their associated risk factors.^4^ In contrast, overall rates of cancer, low birth weight and infant mortality, and years of life lost in the USA all exceed the median values for countries in OECD. Life expectancy is lower.^4^ Interestingly, most of the differences in mortality between the USA and high-performing countries such as France and Japan are the result of deaths that occur before the age of 50 years.^4^

The relatively high costs and poor outcomes that characterize the performance of the United States’ health system are the result of many factors. These factors include poverty, a lack of universal health coverage, a general lack of focus on primary care and public health, high rates of accidents, violence and teenage pregnancy, and poor health behaviours – e.g. poor diets and an overreliance on automobiles for travel – that lead to obesity and lack of fitness.^4^

## Context for reform

The health system differs markedly from its European counterparts. Historically, it has eschewed central health planning and financing. The system originally developed largely through the private sector, with regulation of the public sector carried out at state – rather than federal – level. This less centralized approach generated a pluralistic system in which people may be covered by schemes resembling (i) the public single-payer system of the United Kingdom’s National Health Service – e.g. the Veterans Health Administration, (ii) statutory European social insurance – e.g. Medicare for the disabled and those older than 64 years, (iii) employer-sponsored private insurance; or (iv) individually-purchased private insurance policies (Box 1).

Box 1 **Major payers in the United States of America’s health-care system**

Currently, 48% of health-care expenditure comes from public payers, 40% comes from private payers and 12% is out-of-pocket payments by patients.

The public purchasers of health care – primarily Medicare and Medicaid – cover approximately 30% of the population.

**Medicare**

- Residents older than 64 years, the disabled and those with end-stage renal disease (about 50 million people) are covered by Medicare.
- Medicare is the largest public purchaser of health care and is funded by a combination of payroll taxes, federal tax revenues and enrolee premiums and patient cost-sharing requirements.

**Medicaid and the Children’s Health Insurance Program (CHIP)**

- In 2013, before the main provisions of the Affordable Care Act were implemented, Medicaid covered about 59 million residents and the Children’s Health Insurance Program covered about 6 million children.
- Both programmes are state-administered and historically have covered poor mothers and their children.
- Medicaid also covers disabled adults and, along with Medicare, the low-income elderly who are often referred to as the dual-eligibles. Most state programmes cover the costs of long-term care for individuals who have used up all of their own incomes and assets.
- Medicaid is funded, via a federal–state matching programme, using general federal and state revenues. The federal share, which varies from 50% to 74%, is inversely related to the per capita income of the state.
- As a result of the Affordable Care Act’s expansion of income eligibility for low-income families and childless adults, enrolment in Medicaid is expected to increase by about a third by 2020.

**Other public payers**

Three other public payers are funded by general federal revenues:

- Veterans Affairs, for military veterans;
- Tricare, for active duty military personnel and their families;
- Indian Health Services, for indigenous residents.

**Private payers**

- Most residents – including those with employer-sponsored health insurance, those with individual private insurance and the uninsured – are considered private purchasers of health care. Private insurance falls predominantly into three categories known as health maintenance organizations, preferred provider organizations, and high-deductible health plans.

**Employer-sponsored health insurance**

- More than 90% of residents who have private insurance – approximately 150 million people – have obtained it through an employer.
- Such insurance is funded by a combination of employer and employee premiums and employee cost-sharing requirements.

**Individual private health insurance**

- About 10% of employees – approximately 15 million people – have individually purchased health coverage.
- Individual private health insurance is funded entirely by premiums paid by enrolees and patient cost-sharing requirements.
- Private health insurance is expanding under the Affordable Care Act, with the formation of health insurance exchanges.

**The uninsured**

- In 2012, 47 million people in the United States of America younger than 65 years – approximately 18% of that age group– were uninsured.
- Young adults, minorities and those with low household income are particularly likely to be uninsured.
- As a result of the Affordable Care Act, the number of American adults younger than 65 years who are uninsured is expected to fall to 31 million by 2020.

The USA is currently the only high-income country without nearly universal health-care coverage. Attempts to achieve universal health coverage have been made since the 1940s but – apart from the development of Medicare and Medicaid (which provides coverage for the poor, near-poor residents, including children, pregnant women, parents, seniors and individuals with disabilities) in 1965 – little progress had been made before the implementation of the ACA. The ACA will not bring about universal coverage; it is expected that only about half of the residents who are currently uninsured will ultimately obtain insurance. However the Act establishes a requirement that nearly all legal residents should obtain coverage.

In 2012, two years before implementation of the ACA’s major provisions, 56% of residents younger than 65 years had health-care coverage via their employers. Six per cent purchased private individual health insurance, and about 21% relied on Medicaid.^7^ In general, individually-purchased private health insurance policies are very expensive because they are not subsidized by employers and because the insurers cannot take advantage of the economies of scale associated with the employer-provided policies. Due to various eligibility restrictions and variation in state laws, only about half of the poor were covered by Medicaid in 2012.^8^

## The Affordable Care Act

The ACA became law in 2010, with many of its important provisions going into effect in 2014 (Box 2, Box 3). The timing of the ACA and the new major government programme that it initiated was particularly challenging, since debate about the legislation occurred during a major economic recession.

Box 2 **Key provisions of the Affordable Care Act**

The Affordable Care Act came into effect in January 2014. The Act’s key provisions are the following.

**Expansion of private insurance coverage**

- Subsidies – on a sliding scale – to aid uninsured individuals and families in the purchase of required private health insurance coverage through so-called health-care exchanges. Subsidies are provided to individuals and families with incomes below 400% of the federal poverty level. In 2014, the federal poverty level was 11 670 United States dollars (US$) for an individual and US$ 23 850 for a family of four.
- An individual mandate requiring that all residents and documented immigrants have health insurance coverage. Under most circumstances, failure to have coverage results in a financial penalty that – when the phase-out period ends in 2016 – will be US$ 695 per individual, US$ 2085 per family or – if greater – 2.5% of income. Enforcement will be challenging, however. The main method of enforcement is for the federal government to reduce a person’s annual income tax refund. The federal government cannot put a lien on wages or financial assets.
- The establishment of health insurance exchanges selling private insurance policies. Individual states can establish such exchanges. Residents of a state that does not establish an exchange can purchase health coverage from a federal exchange. All exchanges must offer benefit packages that cover 10 essential health benefits (Box 3), although the exchanges have authority over many of the details. Uninsured individuals, families and small businesses can purchase insurance coverage on these online exchanges – often with the subsidies noted earlier.
- Private insurers selling through the exchanges cannot reject an applicant due to health status or charge more to those with pre-existing medical conditions than to other applicants. Premiums can vary based on age, smoking status and geographical location. No annual or lifetime-limits can be placed on the value of insurance coverage.
- Insurers must either return 80% of premiums in the form of health benefits or provide policy-holders with rebates.

**Public insurance coverage: Medicaid**

- As drafted, the Affordable Care Act required that Medicaid coverage be expanded to everyone with an income below 138% of the federal poverty level. The federal government would pay 100% of the associated costs for the first few years and then 90% subsequently. However, as a result of a ruling of the Supreme Court, such expansion of Medicaid eligibility was made optional at state level. Just over half of the country’s states expanded Medicaid coverage during 2014.

**Public insurance coverage: Medicare**

- The Affordable Care Act specified that, within the Medicare programme, preventive services will be covered without co-payment from the patient.
- Over time, the coverage gap for prescription drug coverage – the so-called doughnut hole – will be removed.
- Medicare Advantage plans – e.g. for managed care – will experience reductions in their capitation rates because of evidence that, on average, payments for such plans exceed their costs. The plans achieving high and low scores for quality will be given bonuses and financial penalties, respectively.
- An Independent Payment Advisory Board will be formed to make recommendations to contain costs if growth in fee-for-service Medicare costs exceeds any corresponding growth in the gross domestic product by more than 1%. However, such recommendations can be overridden by Congress.

**Employers**

- Large employers must either offer health insurance – by 2015 if they have at least 100 employees and by 2016 if they have 50–99 employees – or face a penalty.
- Smaller employers do not have to provide health coverage but their employees are still subject to the individual mandate. Some small employers will receive tax credits if they offer such coverage.
- The so-called Cadillac tax will be imposed on health insurance policies that are very expensive.

**Health-care providers**

- Health-care providers who choose to organize into accountable care organizations have the opportunity to share any savings they receive from Medicare and perhaps, eventually, from other payers.
- Experiments are to be conducted regarding moving away from pure fee-for-service to a programme of bundled service payments.
- Scholarships and loans are being offered to encourage more primary care physicians to work in underserved rural and urban areas, as well as various programmes to train and employ more nurses.

**Consumers**

- Individuals and families with high annual incomes – e.g. above US$ 200 000 and above US$ 250 000, respectively, in 2013 – face higher taxes on unearned and investment income and must pay higher payroll taxes to finance Medicare.

Box 3 **Essential health benefits to be covered by insurers in the exchanges**

All private health insurance plans offered in the exchanges will offer the same set of essential health benefits, which must include:

- ambulatory patient services;
- emergency services;
- hospitalization;
- maternity and neonatal care;
- mental health and substance-use disorder services, including behavioural health treatment such as counselling and psychotherapy;
- prescription drugs;
- rehabilitative services and devices;
- laboratory services;
- preventive and wellness services and chronic disease management;
- paediatric services.

The ACA is much more than just a health insurance law. It touches on almost every aspect of the delivery of the health service and was designed to encourage more primary care, to promote a greater focus on quality and prevention, and to encourage doctors, hospitals and other providers to coordinate care through new entities called accountable care organizations (Box 4). Under the Act, health-care coverage should be increased by (i) the introduction of state-level insurance exchanges, which provide an online market for individuals and small businesses to purchase health insurance coverage (Box 5). Most of these exchanges are currently being administered by – or are in partnership with – the federal government, with substantial income-related tax subsidies; (ii) expansion of the existing Medicaid programme to everyone with income below 138% of the poverty threshold, in those states that have chosen to expand Medicaid eligibility; and (iii) the requirement that, by 2015 or 2016, a firm with at least 50 employees offers and helps pay for its employees’ health insurance. For those who earn too much to qualify for Medicaid but earn no more than four times the standard threshold that indicates poverty – e.g. individuals and families of four that earned up to US$ 47 000 and US$ 95 000, respectively, in 2014 – subsidies are provided on a sliding scale.

Box 4 **Accountable care organizations: an incentive-based experiment with important lessons for Europe**

**Overview**

Accountable care organizations (ACOs) are designed to provide coordinated care of good quality and to control expenditures, in a fee-for-service environment. Although these are associations of providers, their use is being stimulated by public and private insurers. The Affordable Care Act encourages the formation of such organizations as part of the Medicare programme but they are also being used, selectively, by other payers.

ACOs are required to have primary care providers but often also include hospitals and specialists. Most are based in large metropolitan areas and most are sponsored by physician-led groups. They work with insurers to develop reimbursement schemes that provide incentives to provide efficient, high-quality care to the population that they serve. Under the Medicare Shared Savings Program that was established by the Affordable Care Act, ACOs receive financial rewards if they are able to both provide high-quality care and control costs. To determine these financial awards, the performance of the ACOs is compared against benchmark costs. These costs are initially based on the mean Medicare inpatient and outpatient expenditures – in the three years before the formation of the ACO – for each beneficiary assigned to the ACO. The benchmark costs are updated annually. Each ACO must achieve quality standards in four overall areas: (i) patient and caregiver experiences, (ii) care coordination and patient safety, (iii) preventive health, and (iv) populations with chronic diseases.

In December 2013 there were approximately 366 ACOs in the United States of America, which, together, were serving about 15% of Medicare beneficiaries. Experiences with regard to the organization of integrated care and bundled payments – both positive and negative – are accumulating quickly.

**Concerns about ACOs**

ACOs face several challenges in providing high-quality cost–effective care in a fee-for-service setting. First, because ACOs generally rely on highly integrated systems, the consolidation of providers could lead to monopolistic pricing power that could raise costs. Second, there are concerns that, as a result of the financial incentives available, ACOs will put so much pressure on providers that quality could suffer. Finally, there is little evidence that independent providers who are linked together mainly through reimbursement incentives will be able to provide the same quality and continuity of care as health maintenance organizations that oversee the entire patient-care process.

Early experiences with the ACOs have been mixed. One model that received a great deal of attention was the Pioneer ACO Model, which included 32 ACOs from across the country – including some of those serving both Medicare and privately insured patients. In their first year, 18 of the 32 ACOs generated savings but the others generated losses. The indicators of quality were generally good. However, nine of the 32 ACOs chose to drop out of the model, which leads to questions about the model’s sustainability.

Box 5 **Health insurance exchanges: hardly a new idea**

A health insurance exchange is more than an online web portal to purchase insurance. It also requires the organization of several regulatory functions that apply to the private insurance market. An exchange typically provides several insurance plans that offer a minimum list of essential benefits, flat rate – community-rated – premiums that are not based on health status, and tax subsidies for individuals on relatively low incomes. Further components often include the quality rating of plans, enforcement of insurance mandates, compensation payments to insurers with high-cost enrolees and regulatory oversight.

Under the Affordable Care Act, states have received important latitude in how they set up their exchanges. By January 2014, 17 states had opted to develop and manage their own exchanges, 27 states had decided to rely entirely on a federal exchange and the remaining states were developing an exchange jointly with the federal government. The idea behind exchanges is not new; similar systems have been operational in Switzerland since 1996, in the Netherlands and the state of Massachusetts since 2006. One feature that distinguishes the exchanges in the United States of America from some of those in Europe is that, in the former, premiums can vary by age and smoking status.

In the exchanges, premiums may be age-related but the highest age-based premium may be no more than threefold higher than the lowest. The latter restriction means that the youngest people on an exchange cross-subsidize the older ones – because older people who are not yet eligible for Medicare spend about five times as much on health care as people in their mid-20s. There is a fear that too many young, healthy residents will choose to pay the penalty and remain uninsured rather than pay the high premiums needed to cover the costs of care of older individuals. Although the charging of higher premiums to self-declared smokers is generally supported by the public, the low-income groups who have the higher prevalences of smoking are also those least able to pay high premiums. It also seems likely that many smokers will falsely declare themselves to be non-smokers to obtain cheaper insurance. Some states have therefore decided not to allow self-declared smokers to be charged higher premiums than self-declared non-smokers. Data from the exchanges in the Netherlands, Switzerland and the state of Massachusetts indicate that, when used alone, exchanges may not be enough to control costs and that reforms in the purchasing market are also needed.^9^

Any individual who purchases health coverage through the insurance exchanges cannot be turned down for coverage and cannot be charged more than other people who are of the same age and live in the same area. The ACA-related change from experience rating to community rating introduces a major shift away from a private actuarial insurance market and should lead to a financial redistribution, at any point in time, from those who are healthier towards those who have a history of costly illness. Many uninsured individuals who do not receive health-care coverage from an employer and who are not poor enough to qualify for Medicaid – or are not otherwise waived from the requirement – are required to purchase private insurance through one of the new exchanges. Otherwise, most such individuals will be required to pay a penalty – although there are some groups exempted from this requirement.^10^ The penalties to be applied in 2014 and 2015 are quite low but will be gradually increased to US$ 695 per person, US$ 2085 per family or 2.5% of income – whichever is the higher – by 2016.

The implementation of the ACA brings the USA closer to meeting resolution 66/288 of the   Sixty-sixth Session of the United Nations General Assembly, which called on countries to “recognize the importance of universal coverage to [enhance] health, social cohesion and sustainable human and economic development” and to “strengthen health systems towards the provision of equitable, universal coverage and promote affordable access to prevention, treatment, care and support related to non-communicable diseases”.^11^ However, the USA is not expected to meet the goal of universal health coverage in the foreseeable future. It has been estimated that about 31 million residents – undocumented workers, those exempted from mandated coverage because it would be unaffordable, those living in states that chose not to expand Medicaid eligibility, and those individuals and families who choose to pay a penalty rather than purchase health insurance coverage – will remain uninsured in 2016.^12^ Nevertheless, for those who do obtain health coverage, the overall philosophy of the ACA would appear to be consistent with resolution 66/288. For example, the Act (i) prohibits insurers from charging higher premiums because of a history of illness, (ii) requires that insurers cover several preventive services – e.g. screening for abnormal blood pressure, high cholesterol, colorectal cancer and depression – free of any cost-sharing requirements, (iii) requires the subsidization of health insurance coverage for those who, although too wealthy to qualify for Medicaid, would otherwise have difficulty affording coverage; and (iv) promotes progressive financing mechanisms such as higher income and payroll taxes on individuals and families who have incomes above certain thresholds – e.g. US$ 200 000 and US$ 250 000, respectively, in 2013.^13^

## Prospects and challenges

Recently we explored the major challenges that the USA’s health system currently faces in the areas of health coverage, expenditure and quality.^14^

### Health insurance coverage

The ACA was promoted as a way of achieving nearly universal health insurance coverage. It was originally estimated that, in 2016, approximately 21 million residents would remain uninsured – compared with an estimated 56 million had the ACA not gone into effect.^15^^,^^16^ However, in June 2012 the Supreme Court ruled that states would not be required to expand Medicaid eligibility. So far, despite the federal government agreeing to pay 100% of the costs of Medicaid expansion for the first 3 years and 90% of the costs thereafter, only 26 of the USA’s 50 states have chosen to expand Medicaid. This has left about half of the otherwise newly eligible residents without Medicaid coverage.

Public opinion surveys have been the source of most of the early estimates of how the ACA has affected rates of health insurance. For example, recent estimates from the Gallup polling organization indicate that the uninsured population fell from 18% in October 2013 – i.e. shortly before the main parts of the ACA were implemented – to 13.4% six months later.^17^

Some of the leaders of the 23 states that have, so far, chosen to not expand Medicaid have argued that a highly indebted government will not be able to meet its promises to subsidize the future state-level costs of such expansion. The remaining 27 states and the District of Columbia, most of which are led by members of the Democratic Party, have expanded Medicaid – along the lines of the ACA – to everyone with income below 138% of the poverty threshold.^18^ Arkansas has received permission from the federal government to expand Medicaid eligibility through enrolment in commercial insurance plans offered through the health-care exchanges – an option that appears politically viable and that is currently being considered by the leaders of a few other states.

The quality of the insurance coverage has to be considered alongside the extent of such coverage. To keep premiums affordable, insurers have instituted hefty cost-sharing requirements and put together narrow provider networks.^19^ Under the most commonly-purchased plans, annual deductibles typically exceed US$ 2000 per insured individual – and deductibles of this magnitude are likely to have a considerable negative impact on utilization. While cost-sharing requirements are generally clear to most buyers, what is less clear to them is the breadth of the provider network. Although precise figures are not available, there is some evidence indicating that many insurers have kept their rates down by avoiding high-cost providers, including some prestigious hospitals.^20^

### Expenditures

Proponents of the ACA believe that the Act will have several major benefits. They believe that the exchanges will create additional downward pressure on costs – due to increased price competition and choice of policy – and make consumers more cost and quality conscious – by making it easier for them to understand and compare health insurance options. Consumers in several European countries have also been given a larger role in the choice of plans or providers – or both – via websites. Examples include the United Kingdom’s NHS Choices scheme and the Dutch *kiesBeter* or “Choose Better” scheme.

There is currently discussion about whether the USA has already bent the cost curve since, during the past 3 years, growth in medical expenditure has been at historically low levels.^21^ Such low levels may be attributable not only to several provisions of the ACA – e.g. cuts in payments to Medicare’s managed-care plans, incentives to reduce hospital readmissions and the expansion of accountable care organizations^22^ – but also to the economic recession and higher patient cost-sharing.^23^

The possibility remains that there could still be a substantial rebound in medical expenditure if, for example, new blockbuster drugs are introduced or if the movement towards personalized medicine through genetic testing results in higher spending. Moreover, it may become increasingly difficult to control costs as physician groups and hospitals consolidate to augment their market power in negotiations with insurers.^24^^,^^25^ Such consolidation is likely to rise, as accountable care organizations increase their market share, whereas the market power of individual insurers could decline as more insurers compete in the exchanges.

### Quality

Besides the accountable care organizations, several other quality-improvement initiatives under the ACA come with economic incentives. For example, hospitals in the top quartile in terms of hospital-acquired infections and hospitals with the highest risk-adjusted readmission rates for some common diseases will face reductions in their Medicare reimbursements of 1% and up to 3%, respectively. In this value-based purchasing programme, all hospitals face reductions of 2% in their Medicare reimbursements but the money saved is used, in a budget-neutral manner, to reward the hospitals that perform well in terms of various quality measures.

Quality improvement under the ACA is constrained by the supply of primary care providers as well as by Medicare reimbursements that tend to fall well below the reimbursements paid by private insurers. Access to primary care may be partly alleviated by the establishment of patient-centred medical homes and accountable care organizations and by the greater use of electronic medical records, all of which are designed to create efficiencies and reduce the duplication of services. Medical homes have been defined as a model or philosophy of primary care that is patient-centred, comprehensive, team-based, coordinated, accessible, and focused on quality and safety.^26^ The ACA also promotes the provision of funding to train family nurse practitioners who provide primary care in federally qualified health centres and nurse-managed health clinics.^27^

The Patient-Centered Outcomes Research Institute was established by the United States’ Congress to fund and disseminate evidence-based research. To ensure that clinical rather than financial interests are prioritized, the institute is limited in its use of comparative effectiveness data and cannot use dollars per quality-adjusted life-year in its analyses. Public payers like Medicare are also constrained from using such cost–effectiveness data in their health coverage and reimbursement decisions.^28^ Although these restrictions reflect fears that life-saving but expensive procedures might be rationed as a way to reduce costs, they also hamper policy-makers’ attempts to contain costs in the long term.

### Political sustainability

Since the ACA was signed, there has been strong and unwavering opposition from Republicans in the House of Representatives, who have voted for the Act’s repeal over 40 times. Polling of the general public has consistently indicated that more residents oppose the ACA than support it. Of the 1507 nationally representative residents polled in July 2014, for example, 37% supported the ACA and 53% opposed it.^29^

Despite these observations, many elements of the ACA – e.g. the exchanges, the requirements that prior illness does not affect the obtaining of health coverage or the premium a person pays, and allowing children to stay on their parents’ policies until they reach 26 years of age – are generally popular.^30^ Moreover, millions of individuals have benefited from the subsidies to purchase insurance and from the liberalization of Medicaid’s eligibility rules.

While the ACA will probably be able to withstand any political challenges while President Obama remains in office, this may not be the case in the future. Under the United States’ system of government, the President can veto any bill adopted by Congress. If the Republicans held majorities in both houses of Congress and tried to repeal the ACA, they would most likely have to replace the Act with legislation that eliminated the unpopular individual mandate while retaining the provisions that the general public find attractive – e.g. guaranteed issue, extension of dependent health coverage and the subsidies given to individuals and businesses to support the purchase of private insurance on the exchanges.

Elimination of the individual mandate would be tantamount to a reversion to a system of voluntary insurance. It is not clear, however, how a policy like the ACA could keep premiums in check if residents were not required to purchase coverage. The main concern is that healthier people will shy away from the exchanges and purchase health coverage only after they need it. This could make the premiums for people who need to purchase health coverage on the exchanges prohibitively expensive and result in the so-called premium death spiral. In the latter scenario, which has already occurred in some employer-sponsored health plans in the USA, the annual increase in premiums results in ever-increasing attrition among the plan’s remaining, relatively healthy members until the plan becomes unsustainable.^31^^,^^32^

## Going forward

Although several of its main features – including the health insurance exchanges, Medicaid expansion, accountable care organizations and further oversight of insurance companies and their pricing practices – remain works in progress, the ACA has already had a substantial impact on health care in the USA. However, in the current divisive political climate, any new initiatives to achieve universal health coverage will be difficult to legislate or implement. Furthermore, the ACA did not introduce any of the stringent spending controls found in many European health systems. Maryland is the only state that has embraced uniformly regulated prices and Massachusetts is the only state that has tied growth in health-care spending to the growth of the state’s economy.^33^ In general, the USA has not introduced global budgeting or substantial federal measures to limit the supply of providers and technologies. Whereas other high-income countries have set up institutes to assess the cost–effectiveness of pharmaceuticals and health services and technologies – e.g. the National Institute for Health and Care Excellence in the United Kingdom, the *Haute Autorité de Santé* in France and the Pharmaceutical Benefits Advisory Committee in Australia – the ACA explicitly prohibits the creation of such institutes in the USA. While much is expected from the accountable care organizations, the evidence to date suggests that such organizations have only been moderately successful in their aim of rewarding quality and low cost rather than production.^34^^,^^35^

Perhaps the major challenge facing the ACA in the coming years can be traced back to the utter lack of cross-party political consensus. The performance of the ACA and its resulting acceptability to the American public will be critical to the Act’s future.
